# The clinical picture of ICANS after CAR-T cell therapy in critically ill patients: A retrospective clustering analysis of a multicentre multinational cohort study

**DOI:** 10.1016/j.aicoj.2026.100123

**Published:** 2026-07-25

**Authors:** Ana Vagos-Mata, Pedro Castro, Sandrine Valade, Antoine F Carpentier, Alice Gallo de Moraes, Adel Maamar, Muriel Picard, Anne-Sophie Moreau, Sanjay Chawla, Florent Wallet, Kada Klouche, Victoria Metaxa, Nicolas Boissel, Andry Van De Louw, Amélie Seguin, Djamel Mokart, Boris Böll, Roberta Di Blasi, Catherine Thieblemont, Nahema Issa, Sara Fernandez, Laveena Munshi, Philippe R. Bauer, Michael Joannidis, Gabriel Moreno-Gonzalez, Lara Zafrani, Michael Darmon, Elie Azoulay, on behalf of the Nine-I investigators

**Affiliations:** a Department of Hematology and Bone Marrow Transplant, Hospital Santa Maria, Unidade Local de Saúde Santa Maria and Faculty of Medicine, University of Lisbon, Portugal; b Medical Intensive Care Unit, Hospital Clínic of Barcelona, Institut d’Investigacions Biomèdiques August Pi i Sunyer (IDIBAPS), University of Barcelona, Spain; c Critical Care Department, APHP, Hôpital Saint-Louis, University of Paris, France; d Paris Cité University, Assistance Publique – Hôpitaux de Paris, Neurology Department. Saint-Louis Hospital, Paris, France; e Division of Pulmonary and Critical Care Medicine, Mayo Clinic, Rochester, Minnesota, United States; f Critical Care and Infectious Diseases Department, Rennes University Hospital, and INSERM CIC-1414, Faculté de Médecine, Université Rennes 1, France; g Critical Care Department, Institut Universitaire du Cancer de Toulouse-Oncopole, University Teaching Hospital of Toulouse, France; h Critical Care Department, Salengro Hospital, CHU Lille, France; i Critical Care Medicine Service, Department of Anesthesiology & Critical Care Medicine, Memorial Sloan Kettering Cancer Center, New York, NY, United States; j Department of Anesthesiology, Weill Cornell Medical College, New York, NY, United States; k Critical Care Department, HCL, Hôpital Lyon Sud, University of Lyon, France; l Critical Care Department, Hôpital Lapeyronie, University of Montpellier, France; m Department of Critical Care, King’s College Hospital NHS Foundation Trust, London, United Kingdom; n Hematology Department, Division for Young Adults and Adolescents, Saint-Louis Hospital and Paris Cité University, France; o Division of Pulmonary and Critical Care Medicine, Penn State Health Milton S Hershey Medical Centre, Hershey, PA, United States; p Critical Care Department, Nantes University Hospital, France; q Critical Care Department, Institut P-Calmettes, Marseille, France; r Department of Internal Medicine, Haematology and Oncology, Intensive Care Medicine, Centre for Integrated Oncology Aachen Bonn Cologne Duesseldorf, University of Cologne, Germany; s Paris Cité University, Assistance Publique – Hôpitaux de Paris, Hemato-oncology Department, Division for Lymphoma, Saint-Louis Hospital, Paris, France; t Critical Care Department, Hôpital Saint-André, University of Bordeaux, France; u Interdepartmental Division of Critical Care Medicine, Sinai Health System, University of Toronto, Canada; v Division of Intensive Care and Emergency Medicine, Department of Internal Medicine, Medical University Innsbruck, Innsbruck, Austria; w Intensive Care Unit, Bellvitge University Hospital, Catalan Institute of Oncology L’Hospitalet, Bellvitge Biomedical Research Institute, University of Barcelona, Spain

**Keywords:** ICANS, Symptom clusters, Severe ICANS, Hemato-oncology, ICU, CAR T-cells

## Abstract

CAR T-cell therapy carries significant risks, including ICANS. There is limited data on ICANS presentation and outcomes in patients admitted to ICU and it is still challenging to predict those who will have severe ICANS. This is a pre-planned substudy of an international, multicenter, observational cohort study of 241 CAR T-cell recipients for hematological malignancies, admitted to the ICU, focusing on those who developed ICANS. We aimed for a detailed description of symptoms and hierarchical clustering analysis was used to identify distinct patterns of neurological symptom presentation. ICANS was identified in 100 patients (41.5%), median age of 61 years. Most patients (93%) had non-Hodgkin lymphoma. Severe ICANS occurred in 38% of patients, associated with longer duration of symptoms (*p* = 0.02). On the whole cohort, the median duration of neurological symptoms was 5 (IQR 1–8) days and was also longer for patients with isolated ICANS (median of 16 days (IQR 11–24, *p* = 0.001)). Hierarchical clustering revealed four distinct clusters with minimal symptom overlap: Cluster 1: Mild or no symptoms, primarily confusion (11.2%); Cluster 2: High incidence of confusion (78.9%) and drowsiness (47.4%), with significant risks of seizures (42.1%) and coma (21.1%); Cluster 3: Predominantly tremor (75%) and attention deficit (37.5%); Cluster 4: High incidence of aphasia (70.3%), dysgraphia (37.8%), and disorientation (27%). The clustering of neurological symptoms was statistically significant (*p* < 0.001). This study provides a detailed characterization of ICANS, highlighting distinct patterns of neurological symptoms. These findings could inform clinicians, particularly regarding the risk and timing of ICU admission.

## Graphical abstract

**Figure:**
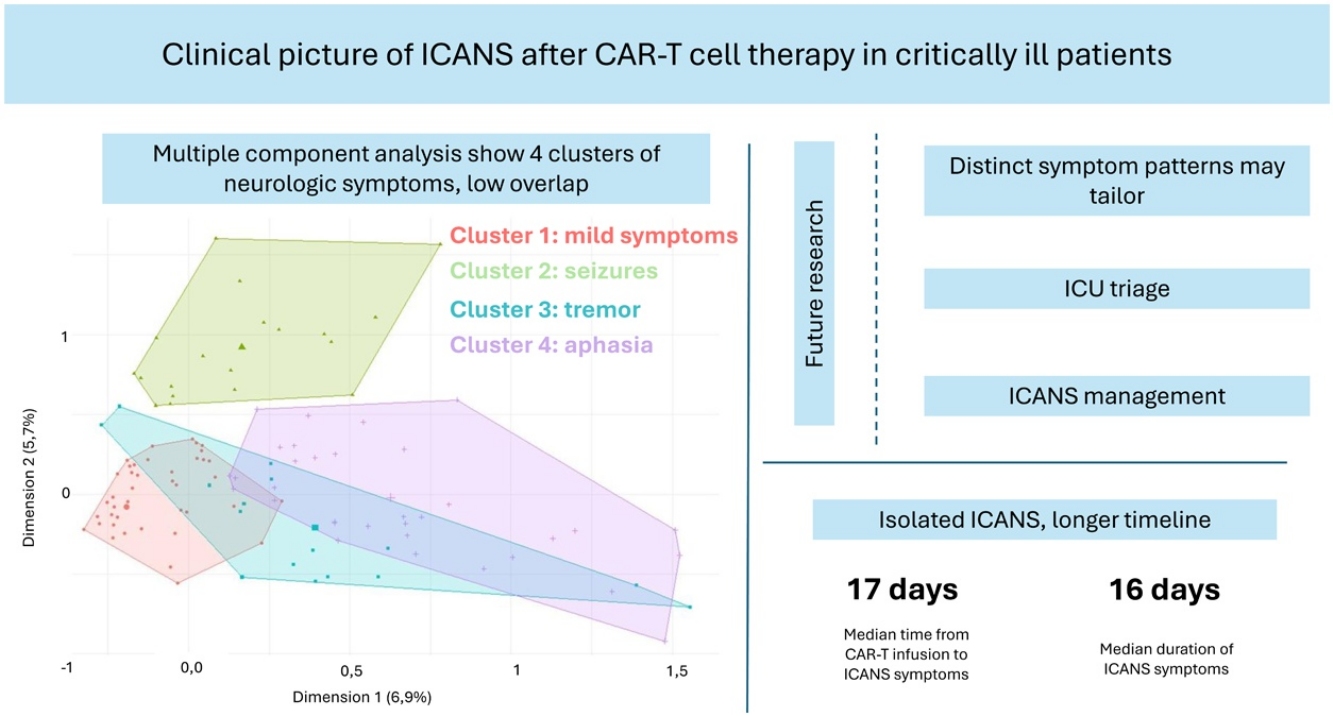

## Introduction

Chimeric Antigen Receptor (CAR) T-cell therapy has revolutionized the treatment of certain hematologic malignancies, offering hope to patients with otherwise refractory conditions [1]. However, this groundbreaking therapy is not without its challenges, as it is associated with several potentially life-threatening toxicities, including Cytokine Release Syndrome (CRS) and Immune Effector Cell-Associated Neurotoxicity Syndrome (ICANS) [2]. While these toxicities have been well-documented in the broader CAR T-cell recipient population, there is a critical need for focused research on those patients who require intensive care unit (ICU) admission due to severe manifestations of these conditions.

ICANS, a complex and often unpredictable syndrome, can present with a wide spectrum of neurological symptoms, ranging from mild confusion to severe encephalopathy, seizures, and cerebral edema [3]. Available evidence suggests the existence of different pathophysiological processes leading to a wide spectrum of severity [4]. Moreover, most but not all ICANS is preceded by CRS [5], suggesting an intriguing role for CRS in the development of ICANS [4].

The management of ICANS in the ICU setting is particularly challenging due to the interplay between neurological deterioration and other concurrent life-threatening conditions, such as severe CRS or sepsis [6,7]. A multivariable model suggested that tocilizumab prescribed to alleviate clinical and biological abnormalities in patient with CRS was associated with the occurrence of neurotoxicity [8], confirming experimental data [9]. Interestingly, in a study assessing the impact of preinfusion electroencephalography (EEG), the presence of abnormalities were risk factor for severe ICANS, suggesting the benefit from preemptive steroid therapy [10]. More recently, another prognostic scoring system identified patients at high risk for severe ICANS (grade ≥ 3) which included female sex, low platelet count, use of axicabtagene ciloleucel and no bridging therapy or stable or progressive disease (SD/PD) after bridging therapy [3]. However, despite the growing use of CAR T-cell therapies, there is limited detailed characterization of the neurological symptoms of ICANS in critically ill patients [[11], [12], [13], [14]], and even less guidance on the clinical thresholds that should prompt ICU admission.

In this study, we provide a comprehensive analysis of neurological manifestations in patients admitted to ICU who have received CAR T-cell therapy. We aim to fill the knowledge gap by offering a detailed description of the neurological symptoms observed in this unique critically ill population. Furthermore, by applying hierarchical clustering techniques, we explore patterns of neurological symptoms and associated outcomes. Our findings seek to inform clinical practice, particularly regarding the timing of ICU admission and management strategies, thus contributing to the optimization of care for this vulnerable patient group.

## Patients and methods

CAR T-cell Toxicity and Sepsis (CARTTAS) is an international (8 countries), multicentre (21 hospitals), observational cohort study established by the Nine-I study group, which is composed of critical care physicians who have extensive experience in the management of critically ill immunocompromised patients. Investigators retrospectively included patients admitted in the ICU after receiving CAR T-cell therapy from Feb 1, 2018 and Feb 1, 2019 and prospectively included patients admitted between March 1, 2019 and Feb 1, 2020. Both patients who were enrolled in clinical trials and those receiving commercially approved products were included. The present cohort analysis relates to all consecutive patients included in CARTTAS study who developed immune effector cell-associated neurotoxicity (ICANS).

Patients were considered eligible if they were adults, had received CAR-T cell therapy 30 days before the development of ICANS, and developed ICANS either before or after ICU admission. ICANS was diagnosed if there were neurologic symptoms after CAR T-cell infusion, with an adequate timing between infusion and symptom onset and if there was no other more plausible aetiology. Clinical judgment, brain imaging exams, electroencephalogram, laboratory workup and lumbar puncture were fundamental steps on differential diagnosis. All patients were evaluated by highly trained physicians.

Patients with mild neurologic symptoms exclusively considered as being consequence of other aetiologies were considered as free of ICANS.

Institutional review board approval was obtained from each institution in accordance with local ethics regulations. Written informed consent was waived from the retrospective part of the study and obtained for all participants in the prospective part of the study.

A standardized electronic case report form was used to collect the variables listed in the tables and figures. The Sequential Organ Failure Assessment (SOFA) score, used to predict ICU mortality based on clinical and laboratory parameters, was calculated within 24 h after admission to intensive care [15]. Sepsis was either clinically or microbiologically documented. Clinically documented infection included a clinical source of sepsis (such as pneumonia, enterocolitis and skin and soft tissue infections) [16]. Performance status was evaluated according with Eastern Cooperative Oncology Group (ECOG) score. Frailty was defined as a clinical state of increased vulnerability before admission to intensive care and was assessed using the Clinical Frailty Scale (CFS), which is scored from 1 (very fit) to 9 (terminally unwell), as previously reported [17]. Cytokine release syndrome was graded based on the ASTCT (American Society for Transplantation and Cellular Therapy) consensus grading system, which comprises the presence of fever, the severity of hypotension and hypoxaemia [18]. The ICANS grading was calculated according to ASTCT guidelines [18]. It was based on the patient’s orientation and ability to name objects, write a sentence and count backwards (CARTOX-10 score), as well as the level of consciousness and presence of seizures, motor findings and/or elevated intracranial pressures or cerebral edema [11]. In patients requiring invasive mechanical ventilation, neurologic assessment before mechanical ventilation was used. For patients enrolled retrospectively, ICANS was graded retrospectively based on the elements that were reported in the medical charts and CARTOX-10 score wasn’t evaluated. Any neurologic symptoms (aphasia, impaired attention, disorientation, confusion, dysgraphia, tremor, headache, amnesia, drowsiness, focal deficits, myoclonia, seizures, coma, agitation, cerebellar symptoms) presenting during the first 24 h of ICU admission, that were reported in the patient’s chart, were collected. The CARTOX-10 score was prospectively calculated for each patient, with and without ICANS, every day by the intensive care clinicians and reported in the medical chart, for the first seven days of ICU admission [8,19]. Resolution of neurologic symptoms was also collected from medical charts. Every seizure classified ICANS as grade 3. Severe ICANS was defined as ICANS grade 3 or 4. Brain imaging, lumbar puncture and electroencephalogram were performed based upon clinician judgment and were analysed by the local radiologist, pathologist or physiologist.

Clinical management was not protocolized and left to every ICU and haematology specialists across centres. All management decisions were at the discretion of the attending physicians, who followed standard practice and local protocols for managing CAR T-cell therapy toxicities. In patients with neurotoxicity, either isolated or associated with CRS, steroid therapy was started with 20 mg/m^2^/day of dexamethasone or high dose (>2 mg/kg) methylprednisolone [8,19].

The primary study objectives were to describe ICANS in critically ill patients and identify clusters of symptoms until the first 24 h of ICU admission.

Continuous variables were described as median and interquartile range and compared using Wilcoxon rank-sum test or the Kruskal-Wallis test; categorical variables were summarized by counts (percent) and compared using exact Fisher test or Pearson’s chi-square test.

We used a two-step clustering approach to reduce the dimensions of the dataset and to perform a hierarchical clustering (namely, hierarchical clustering on principal components). First, we performed a multiple component analysis (MCA) including a set of clinical data (reported in Supplemental Table 2a and b). A hierarchical cluster analysis from the PCA/MCA was used to determine subgroups of patients according to these characteristics. The clustering of patients was performed using Euclidean distance and the Ward agglomerative method. The set of data was complete on analysed data and no imputation method was used. All 241 patients from CARTTAS study were included on this analysis and only symptoms from the first 24 h after ICU admission were used. The medical centre from which each patient came was included in the analysis, to account for any centre effect. All tests were two sided, and p values lower than 5% were considered to indicate significant associations. Analyses were performed using R statistical platform, Version 4.0.2 (https://cran.r-project.org/).

## Results

Among the 241 CAR T-cell recipients admitted to the ICU, ICANS was identified in 100 patients (41.5%, median age of 61 years (IQR 45–67), 57% men). Most patients (93%) had non-Hodgkin lymphoma, while 7% had acute lymphoblastic leukemia. There was no known active CNS disease at the time of CAR T-cell infusion and all patients received lymphodepletion, most with fludarabine and cyclophosphamide. Apart from SOFA score on admission, higher in patients with ICANS (*p* = 0.01), there was no significant differences between patients with and without ICANS, regarding demographic characteristics, laboratory parameters on ICU admission, length of ICU and hospital stay and ICU mortality rate (Supplemental Table 1).

Patients with ICANS (*N* = 100) were generally in good physiological state, with a median ECOG score of 1 (IQR 1–3) and a median Clinical Frailty Scale score of 2 (IQR 2–3). Patients were admitted to ICU a median of 5 days (IQR 4–8) post-infusion, with the first neurological symptoms appearing after a median of 5.5 days (IQR 5.5–9) post-infusion. Symptoms emerged before ICU admission in 27% of patients, on day one in 58%, and during the ICU stay in 15%. Severe ICANS was observed in 38% of patients. The median CARTOX-10 score at ICU admission was 7 (IQR 3–9). CRS was diagnosed in 93 patients, with median grade of 2 (IQR 1–3) (Table 1). Laboratory parameters on ICU admission are described in Table 1.

**Table 1:** Patient’s characteristics at ICU admission.

Characteristics	*N* = 100 (%)
	or Median (IQR)
Age	61 (45–67)
Sex (female)	43 (43)
Comorbidities	
Hypertension	22 (22)
Diabetes	14 (14)
Performance status (ECOG)	1 (1–3)
Clinical frailty scale	2 (2–3)
Underlying malignancy	
Non-Hodgkin B-cell lymphoma	93 (93)
Acute lymphoblastic leukemia	7 (7)
Central nervous system disease	0 (0)
Previous stem-cell transplantation	
None	79 (79)
Autologous	16 (16)
Allogeneic	5 (5)
Lymphodepletion	100 (100)
Time (days) between CAR-T cell infusion and ICU admission	5 (4–8)
Time (days) between CAR-T cell infusion and ICANS onset	5.5 (5–9)
Onset of ICANS	
Before ICU admission	27 (27)
First day of ICU admission	58 (58)
>2 days after ICU admission	15 (15)
ICANS plus cytokine release syndrome	93 (93)
Isolated ICANS	7 (7)
SOFA score on admission	4 (3–6)
SOFA score on admission – neurologic component	0 (0–1)
SOFA score on admission – neurologic component	
0	51 (51)
1	32 (32)
2	9 (9)
3	4 (4)
4	4 (4)
Infection on ICU admission	16 (16)
C-reactive protein (mg/L)	42.5 (11–118)
Platelet count (cells/µL)	57 500 (33 000–110 000)
Neutrophil count (cells/µL)	360 (22–1055)
Ferritin (ng/mL)	1641 (658–3530)
CARTOX-10 on ICU admission	7 (3–9)
Max. CRS grade throughout the ICU stay	2 (1–4)
ICANS Grade	
Maximum throughout the ICU stay	2 (1–3)
Grade 1	30 (30)
Grade 2	25 (25)
Grade 3	29 (29)
Grade 4	4 (4)
Missing	12 (12)
Need for invasive mechanical ventilation	17 (17)
Need for vasopressors	28 (28)
Need for renal replacement therapy	12 (12)

Results are expressed in numbers (%) or median (interquartile range); laboratory values reported at ICU admission.

CAR-T: chimeric antigen receptor t-cell; CFS: clinical frailty scale; CRS: cytokine release syndrome; ECOG-PS: Eastern Cooperative Oncology Group-Performance Status; ICANS: immune effector cell-associated neurotoxicity syndrome; SOFA: sequential organ failure assessment.

There were 233 neurological events reported among patients with ICANS, with most patients (79%) having experienced at least two different symptoms. Confusion was present in 51% of patients, followed by drowsiness (40%), aphasia (27%), and dysgraphia (19%) (Supplemental Table 2a and b). Seizures and coma were less common, with incidences of 8% and 4%, respectively. Compared to non-severe ICANS, patients with severe ICANS had a significantly longer duration of symptoms (6 vs. 2 days, *p* < 0.001) (Supplemental Table 3). Strikingly, patients with isolated ICANS had higher SOFA scores on admission, lower platelet count, a longer interval between CAR-T cell infusion and ICANS onset (17 vs. 5 days, *p* = 0.002) and a longer duration of neurological symptoms (16 days vs. 3.9 days, *p* = 0.001) (Supplemental Table 4).

**Fig. 1:**
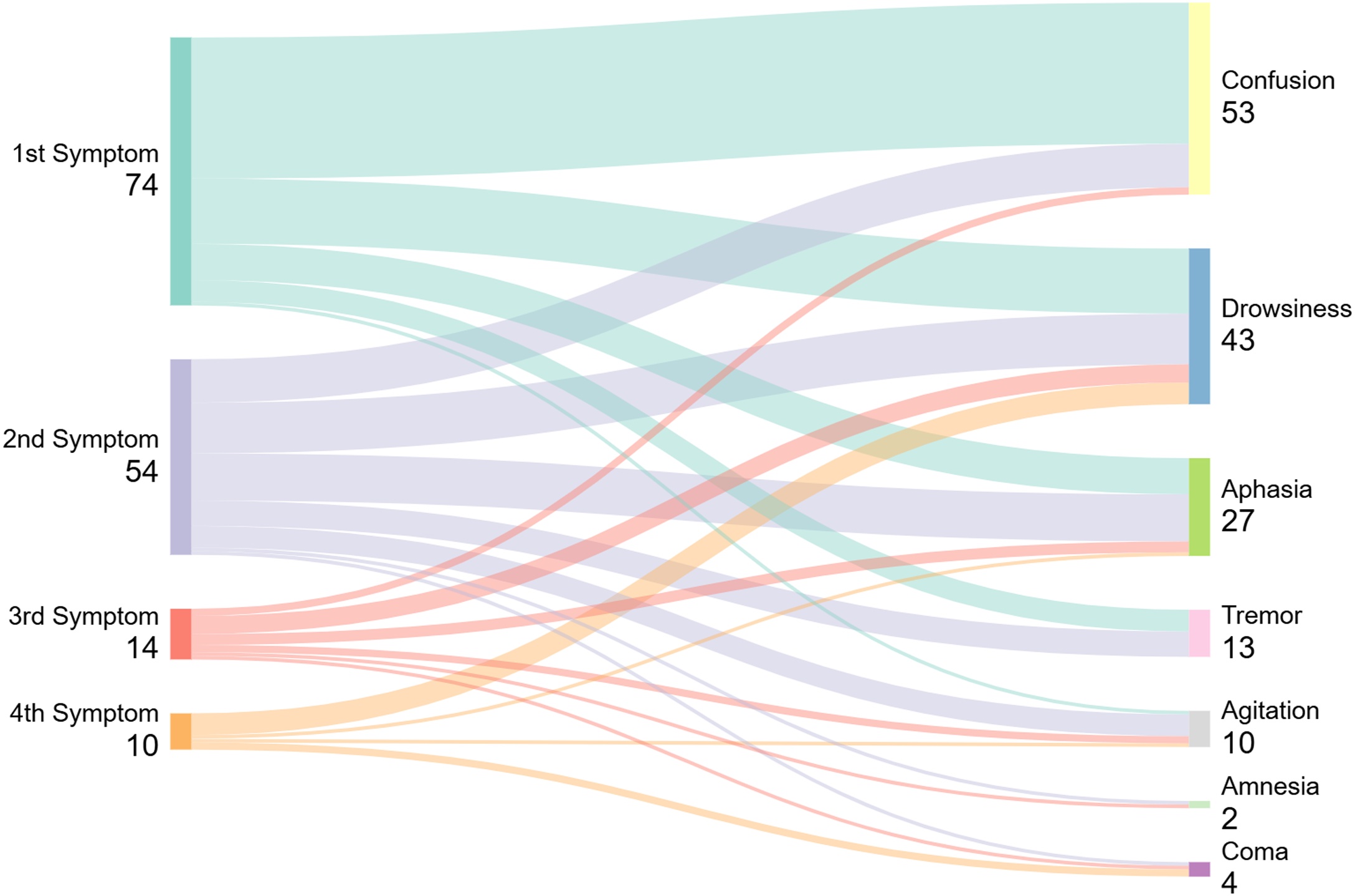
Order of appearance of some neurologic symptoms varied significantly. This figure illustrates how symptoms presenting as first, second, third or fourth symptoms (on the left) are enriched by specific neurologic manifestations (on the right). Only symptoms with significantly different distribution are presented. Symptoms are expressed in numbers (N).

The sequence of symptom onset varied significantly among some neurological manifestations. Confusion, drowsiness, aphasia, and tremor appeared earlier, often as the first or second symptoms (*p* < 0.001 and *p* = 0.001). In contrast, agitation was more frequently noted as a second symptom (*p* < 0.001), while amnesia and coma generally emerged later, typically as second or third symptoms (*p* = 0.001 and *p* = 0.009). Fig. 1 illustrates this flow of symptoms’ appearance.

At ICANS presentation, imaging was performed in 90 patients, every of them but 8 being considered normal. Follow-up imaging in 52 patients (a decision at the discretion of the attending physician) revealed new alterations in 6 patients whose initial imaging was normal: small infarct areas, venous sinus thrombosis, diffuse hyperintensity of cerebral white matter and other MRI (magnetic resonance imaging) signal abnormalities. Also, electroencephalograms (EEGs) were conducted in 70 patients and lumbar punctures in 48 patients (Suplemental Table 5). Unsurprisingly, the presence of epileptiform discharges was associated with severe ICANS (*p* = 0.01).

Prophylactic anti-seizure therapy was administered to 59% of patients. Therapeutic anti-seizure treatment was given to 22%: 8% had seizures, 5% had severe ICANS without seizures, 3% had epileptiform discharges on EEG, and 6% received it at the physician's discretion for grade 1 or 2 ICANS. The ICANS therapeutic plan included steroids in 91% of patients, primarily dexamethasone (72%). Only 25% of patients received steroids alone, while 75% also received anti-IL6 therapy for concomitant CRS (Suplemental Table 6). Anti-IL6 therapy administration did not correlate with ICANS severity (*p* = 0.371)

Median SOFA at ICU admission was 4 (IQR 3–6). Throughout the ICU stay, 17% of patients needed invasive ventilation, 28% needed vasopressors and 12% needed renal replacement technique. Median duration of neurological symptoms of 4.75 (IQR 1.3–8) days. ICU mortality was 7%, and hospital mortality was 17%. Median ICU length of stay was 4 days (IQR 2–7).

**Fig. 2:**
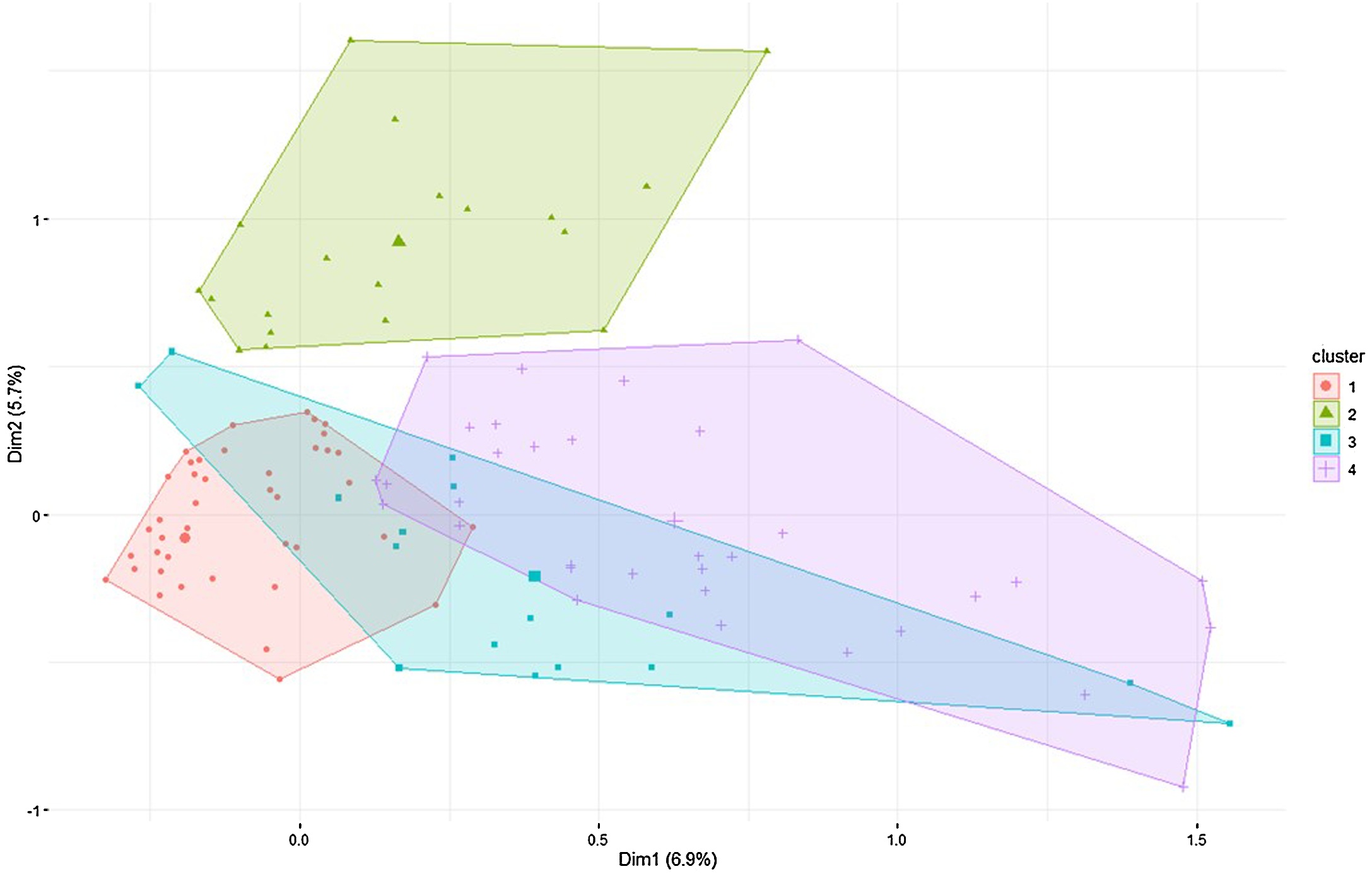
Hierarchical clustering on multiple components analysis: four different clusters were identified. Individuals’ visualization and coloration is set according to the cluster they belong to.

To identify patterns of neurological symptom presentation until the first 24 h of ICU admission, we conducted a hierarchical clustering analysis on the entire ICU cohort (*n* = 241), revealing four distinct clusters with minimal symptom overlap and four main ICANS presentation patterns (Fig. 2, Supplemental Fig. 1). Cluster 1 is characterized by mild or no symptoms, with 90.5% of patients asymptomatic, 11.2% experiencing confusion, and 4.7% drowsiness. Cluster 2 exhibited high incidences of confusion (78.9%), drowsiness (47.4%), agitation (42.1%), with significant risk of seizures (42.1%), and coma (21.1%). Notably, all seizure and coma cases were in this cluster. Cluster 3 predominantly featured tremor (75%), attention deficit (37.5%), and amnesia (12.2%). Cluster 4 was marked by a high incidence of aphasia (70.3%), dysgraphia (37.8%), and disorientation (27%). Tremor and aphasia were associated with clusters 3 and 4, respectively, as these symptoms did not appear on other clusters. Importantly, no patients in clusters 3 or 4 developed seizures or coma during ICU stay. Symptom distribution across clusters was statistically significant (*p* < 0.001), except for cerebellar symptoms, which were rare.

Cluster 2 had fewer patients with diffuse B-cell lymphoma and more with acute lymphoblastic leukemia, while clusters 3 and 4 were enriched with non-Hodgkin B cell lymphoma patients (*p* = 0.05). CRS was highly prevalent in clusters 3 and 4 (93.8% and 94.96%), but less so in cluster 2 (68%), where severe symptoms like seizures and coma were common (*p* = 0.02). Interestingly, most patients with isolated ICANS (6 out of 7) were included in cluster 2. There was no significant difference in ICU length of stay, neither on ICU mortality across clusters (*p* = 0.29 and *p* = 0.54 respectively), although ICU deaths were rare (Table 2). Mechanical ventilation was required more often in cluster 2 than in other clusters (*p* = 0.03).

**Table 2:** Symptoms distribution and patients’ characteristics across clusters.

Clusters		1	2	3	4	P Value
		No or mild symptoms	Confusion, agitation, seizures	Tremor, attention deficit	Aphasia, drowsiness	
Patients per cluster (N)		169	19	16	37	
Patients with ICANS (N)		30	19	15	36	
Characteristics						
Confusion		19 (11.2)	15 (78.9)	6 (37.5)	11 (29.7)	**<0.001**
Drowsiness		8 (4.7)	9 (47.4)	4 (25.0)	19 (51.4)	**<0.001**
Aphasia		1 (0.6)	0 (0.0)	0 (0.0)	26 (70.3)	**<0.001**
Dysgraphia		0 (0.0)	1 (5.3)	4 (25.0)	14 (37.8)	**<0.001**
Tremor		0 (0.0)	1 (5.3)	12 (75.0)	0 (0.0)	**<0.001**
Disorientation		4 (2.4)	1 (5.3)	0 (0.0)	10 (27.0)	**<0.001**
Focal deficits		0 (0.0)	0 (0.0)	2 (12.5)	8 (21.6)	**<0.001**
Headache		1 (0.6)	3 (15.8)	1 (6.2)	4 (10.8)	**<0.001**
Myoclonia		0 (0.0)	3 (15.8)	0 (0.0)	1 (2.7)	**<0.001**
Seizures		0 (0.0)	8 (42.1)	0 (0.0)	0 (0.0)	**<0.001**
Agitation		0 (0.0)	8 (42.1)	0 (0.0)	2 (5.4)	**<0.001**
Cerebellar symptoms		2 (1.2)	0 (0.0)	1 (6.2)	1 (2.7)	0.412
Amnesia		0 (0.0)	0 (0.0)	2 (12.5)	0 (0.0)	**<0.001**
Coma		0 (0.0)	4 (21.1)	0 (0.0)	0 (0.0)	**<0.001**
Attention deficit		0 (0.0)	0 (0.0)	6 (37.5)	1 (2.7)	**<0.001**
None		150 (88.8)	0 (0.0)	0 (0.0)	0 (0.0)	**<0.001**
Gender (Female)		67 (39.6)	8 (42.1)	6 (37.5)	16 (43.2)	0.97
Age (years, median [IQR])		58 [43−65]	57 [27−67.5]	59 [48.5−67.5]	63 [52−67]	0.34
Underlying malignancy						**0.05**
	ALL	27 (16)	4 (21.1)	0 (0.0)	0 (0.0)	
	Diffuse B cell lymphoma	138 (81.7)	15 (78.9)	16 (100.0)	37 (100.0)	
	Multiple Myeloma	4 (2.4)	0 (0.0)	0 (0.0)	0 (0.0)	
CRS (yes)		131 (77.5)	13 (68.4)	15 (93.8)	35 (94.6)	**0.03**
Need for mechanical ventilation		7 (4.1)	4 (21.1)	2 (12.5)	3 (8.1)	**0.03**
Need for vasopressors		50 (29.7)	4 (21.1)	3 (18.8)	8 (21.6)	0.57
Time from CAR-T infusion to first symptoms		5 [4–9]	6 [4–10]	5 [4–9]	6 [4–8]	0.92
CARTOX-10 on ICU admission		8 [6–9]	3 [1–9]	8 [8,9]	5 [2–8]	**0.02**
Duration of neurological symptoms		3 [1–7]	5 [3–10]	5 [3–7]	5 [2–10]	0.73
Length of ICU stay		3 [2–6]	6 [2–9]	3 [2–4]	4 [1–6]	0.29
ICU mortality (yes)		12 (7.1)	1 (5.3)	0 (0.0)	1 (2.7)	0.54
Hospital mortality		26 (15.4)	5 (26.3)	2 (12.5)	3 (8.1)	0.34
Day-90 mortality		39 (23.1)	5 (26.3)	3 (18.8)	7 (18.9)	0.90

Results are expressed in numbers (%) or median (interquartile range). All comparisons were unadjusted using Kruskal-Wallis test and Fisher exact test.

Two patients without ICANS were included in cluster 3 and 4. Both had either drowsiness or confusion, one at early phase of documented bacterial infection and the other during disease progression.

ALL: acute lymphoblastic leukemia; CAR-T: chimeric antigen receptor T cell; CRS: cytokine release syndrome; ICU: intensive care unit; ICANS: immune effector cell-associated neurotoxicity syndrome; MM: multiple myeloma.

Numbers in bold refer to statistically significant p values.

## Discussion

In this study, we provide a detailed analysis of ICANS in critically ill patients who received CAR T-cell therapy, focusing on symptom patterns, severity, and associated outcomes. Our findings highlight the complexity of ICANS management in ICU, where the interplay between neurological deterioration and other concurrent life-threatening conditions, such as CRS and infections, creates significant challenges.

One of the key findings from our study is the identification of distinct neurological symptom clusters through hierarchical clustering analysis. This approach allowed us to uncover four main ICANS presentation patterns in the ICU, with each cluster displaying a unique combination of symptoms that reflect ICANS presentation. Notably, Cluster 2, characterized by a high incidence of severe symptoms such as seizures and coma, was associated with a lower prevalence of CRS compared to Clusters 3 and 4, where CRS was almost the rule. This finding suggests that the presence of CRS may not always correlate with the most severe neurological manifestations of ICANS. Furthermore, until the first 24 h of ICU stay, seizures and coma were uniquely identified in patients belonging to Cluster 2, also enriched with patients with confusion, highlighting that some patterns of symptoms may have a stronger association with life-threatening ICANS. Hence, none of the patients with tremor or aphasia as main feature of ICANS developed life-threatening ICANS features, such as seizure and coma.

To the best of our knowledge, this is the first descriptive analysis of ICANS demonstrating clusters of symptoms and its association with risk of clinical deterioration. This study was not powered to assess prognosis based on clusters, neither to shape medical decisions based on clusters. However, these findings may raise other important questions and help in clarifying patient management individualization.

First, as regard to triaging, our study suggests that patients with initial isolated drowsiness, tremors or aphasia (clusters 1, 3 and 4 respectively, in this study), are at low risk of neurological deterioration, suggesting that systematic ICU admission may not be required at sole identification of ICANS. In addition, since clinical course seems to be more severe in patients with confusion, agitation and seizure (cluster 2 in this study), future trials may assess benefits of more aggressive management in these patients, including early ICU admission and aggressive treatment, for example using high dose steroids or Anakinra. Unfortunately, these therapeutic proposals are based upon current literature rather than in data from our study. ICANS management, in the current study, was too homogeneous to allow any identification of specific response to treatment, according to clusters.

Some questions remain unanswered regarding reliability and external validity of our clustering analysis and its ability to allow tailoring of therapeutic strategy. Nevertheless, this proof-of-concept study suggests identifying clinical clusters is feasible. Future studies addressing reliability in different settings and clinical relevancy are required to confirm and expand our findings.

Our study also identified a significantly longer duration of neurological symptoms in patients with severe ICANS. It further underscores the need for prompt and aggressive management in this subgroup to mitigate the risk of prolonged ICU stays and associated complications [20].

ICANS pathophysiology is not completely understood, but most evidence highlights that endothelial activation, blood brain barrier disruption, the diffusion of cytokines and transmigration of CAR T-cells into the CSF and central nervous system (CNS) predispose to neurologic toxicity [4,21]. Interestingly, our analysis revealed that patients with isolated ICANS had distinct clinical characteristics. These patients presented with significantly higher SOFA scores at admission, lower platelet counts, and a longer interval between CAR T-cell infusion and the onset of neurological symptoms. Unsurprisingly, CRP levels were lower in patients with isolated ICANS, suggesting a lower systemic decompartmentalization in isolated ICANS.

Besides, isolated ICANS was associated with a significantly longer duration of symptoms compared to patients with combined ICANS and CRS. This finding suggests that the absence of CRS does not necessarily imply a milder course of ICANS and highlights the importance of considering isolated ICANS as a potentially severe and prolonged condition requiring intensive management.

Imaging and EEG findings in our cohort also provided valuable insights into the neurological manifestations of ICANS. While imaging abnormalities were relatively uncommon, follow-up imaging in patients with initially normal scans revealed new alterations in a subset of patients, indicating the dynamic nature of ICANS. EEG findings varied widely, with diffuse slowing and encephalic dysfunction being the most common abnormalities and the presence of epileptiform discharges, a surrogate for seizures, strongly correlating with severe ICANS. These results suggest that while imaging and EEG are useful tools in the evaluation of ICANS, their findings should be interpreted in the broader clinical context, considering the patient's overall neurological status and other laboratory parameters [10].

The therapeutic strategies employed in our cohort reflected the current standard of care for ICANS, with steroids being the mainstay of treatment. Our data indicates that most patients received dexamethasone, with doses tailored to the severity of ICANS. The addition of anti-IL6 therapy happened in the earlier period of the study, while limited data were available on its potential toxicity [8]. Despite these interventions, the mortality rates in our cohort, although relatively low, underscore the seriousness of ICANS in critically ill patients and the need for continued research into optimizing therapeutic approaches. For instance, the use of anakinra, an interleukin 1 inhibitor, was first dedicated to steroid-refractory ICANS [22], and is now given earlier in high risk patients, or in severe ICANS [23,24]. At the time of the present study, anakinra was not used. Prophylactic studies of anakinra to mitigate ICANS are ongoing [25]. We also have to acknowledge that ICU admission rates after CAR-T cell treatment have been declining over the last years [26]. Despite a change in trends, our study describes in detail different patterns of ICANS presentation, which is useful regardless of the clinical setting.

Our study has several limitations. First, its design and lack of independent validation may have introduced biases in terms of exhaustivity of symptoms reporting on its timing and order of appearance, as well as on grading of ICANS. Also, due to the retrospective nature of the study, some neurologic symptoms may have been mis-labelled. Validation studies are needed to confirm our findings and delineate more clearly the time course of symptoms, its’ definition, and ICANS severity according to initial symptoms presentation and accounting for potential confounders, such as septic encephalopathy. Additionally, the heterogeneity of the patient population, with varying underlying malignancies, treatment regimens and CAR-T constructs, may limit the generalization of our findings. In this line we were unable to assess influence of CAR-T construct on ICANS incidence, neurological symptoms or subsequent clusters. That is an important limitation, since ICANS incidence differs, according with the type of construct. However, not knowing these data doesn’t prevent the analysis of the neurologic symptoms and the identification of four clusters of ICANS presentation on the first 24 h of ICU admission. It prevents us from drawing associations between the CAR T-cell construct infused and cluster allocation. Regardless of the type of construct, this is the first study suggesting that clinical presentation of ICANS may help guiding patients’ management and ICU triage.

Despite including patients with acute lymphoblastic leukemia, multiple myeloma and diffuse large B cell lymphoma, most patients who experienced ICANS had diffuse large B cell lymphoma (93%), so our results may only be applicable to this subgroup of patients. Moreover, even though patients were treated in expert centres and according to protocols, variability in ICU admission criteria are reported in this series of critically ill patients.

In conclusion, this study provides important insights into the neurological manifestations of ICANS in critically ill hemato-oncology patients, highlighting distinct symptom patterns. These findings may have significant implications for the management of ICANS and ICU triage, particularly in identifying the patients who are at risk for life threatening manifestations of ICANS, and need an early referral for ICU admission, and those who most probably won’t need ICU care. Future research should focus on prospective studies, with careful baseline disease status characterization and a longer time frame of symptom report, to validate these findings, assess its prognostic value, and explore novel therapeutic interventions to improve outcomes for patients with ICANS.

## CRediT authorship contribution statement

EA was principal investigator; EA, MD, AVM designed the study; MD, AVM did the statistical analysis; EA, MD and AVM analysed and interpreted the data and drafted the work. CRF reviewed the data and discussed the findings. All authors approved the protocol, revised the electronic case report form, applied for their local institutional review board, collected informed consent, included patients and their data on the case report form, and contributed to revising the analyses, editing the manuscript and have approved the submitted version. All authors had full access to all data in the study and had final responsibility for the decision to submit for publication. EA, MD and AVM had access to and verified the raw data.

## Ethics approval and consent to participate

Institutional review board approval was obtained from each institution in accordance with local ethics regulations. Written informed consent was waived from the retrospective part of the study and obtained for all participants in the prospective part of the study.

## Consent for publication

Not applicable.

## Financial competing interests

EA has received personal fees and non-financial support from Pfizer, Gilead, Ablynx, Baxter, Alexion and grants from Fisher & Payckle and Merck Sharp & Dohme, outside the submitted work. BB has received personal fees from Johnson & Johnson and Novartis, grants and personal fees from Astellas, Kite/Gilead, Merck Sharp & Dohme and Takeda, outside the submitted work. VM has received personal fees from Gilead, outside the submitted work. MD has received personal fees from Gilead-Kite and Astellas, and grants and personal fees from Merck Sharp & Dohme, outside the submitted work. SV has received non-financial support from Pfizer and personal fees from Gilead-Kite, outside the submitted work. AVM has received personal fees from Gilead and Abbvie, outside the submitted work. All other authors declare no competing interests.

## Funding

Groupe de Recherche Respiratoire en Réanimation Onco-Hématologique.

## Availability of data and materials

The datasets used and analysed during the current study are available from the corresponding author on reasonable request.
