# Supplementary material for: The clinical picture of ICANS after CAR-T cell therapy in critically ill patients: A retrospective clustering analysis of a multicentre multinational cohort study
Source: Ann Intensive Care. 2026 Jul 25;16:100123. doi: 10.1016/j.aicoj.2026.100123 (PMC13464376; doi:10.1016/j.aicoj.2026.100123)
Supplement: Supplementary file 1 [file mmc1.docx]

**Supplemental table 1: Comparison of patients with and without ICANS**

| **N (%) or Median (IQR)** | **ICANS** | **No ICANS** | **P value** |
| --- | --- | --- | --- |
| N | 100 | 141 |  |
| Age (years) | 61 (45 – 67) | 58 (42 – 65) | 0.19 |
| Gender (female) | 43% | 57% | 0.46 |
| SOFA score | 4 (3 – 6) | 3 (2 – 5) | **0.010** |
| ECOG-PS | 1 (1 – 2) | 1 (0 – 1) | 0.50 |
| CFS | 3 (2 – 3) | 2 (2 – 3) | 0.07 |
| CRP (mg/L | 42.5 (11.5 – 118.5) | 40.0 (10.15 – 110.75) | 0.87 |
| Ferritin (ng/L) | 1641.5 (658.75 – 3530.75) | 1421.5 (612.5 – 3144.5) | 0.74 |
| Platelet count (cells/µL) | 57 500 (33 000 – 110 750) | 76 000 (33 500 – 124 000) | 0.27 |
| Neutrophil count (cells/µL) | 360 (22 – 708) | 500 (30 – 1550) | 0.52 |
| Length of ICU stay | 4 (2 – 7) | 4 (2 – 6) | 0.22 |
| Length of hospital stay | 16 (10 – 25) | 14 (9 – 28) | 0.68 |
| ICU mortality rate | 7 (7%) | 7 (5%) | 0.11 |

Results are expressed in numbers (%) or median (interquartile range); laboratory values reported at ICU admission;

CAR-T: chimeric antigen receptor t-cell; CFS: clinical frailty scale; CRP: c-reactive protein; CRS: cytokine release syndrome; ECOG-PS: Eastern Cooperative Oncology Group-Performance Status; ICANS: immune effector cell-associated neurotoxicity syndrome; SOFA: sequential organ failure assessment;

**Supplemental table 2a: Incidence of symptoms**

**(N=100 (%))**

| **Neurological symptoms** | **ICANS cohort (N=100;(%))** |
| --- | --- |
| Confusion | 51 (51) |
| Drowsiness | 40 (40) |
| Aphasia | 27 (27) |
| Dysgraphia | 19 (19) |
| Disorientation | 15 (15) |
| Tremor | 13 (13) |
| Focal deficits | 10 (10) |
| Agitation | 10 (10) |
| Headache | 9 (9) |
| Seizures | 8 (8) |
| Impaired attention | 7 (7) |
| Myoclonus | 4 (4) |
| Cerebellar symptoms | 4 (4) |
| Amnesia | 2 (2) |
| Coma | 4 (4) |
| Results are expressed in numbers (%) |  |

**Supplemental Table 2b: Incidence, relative frequency and order of appearance of the neurological symptoms reported in critically ill patients with ICANS**

| **Symptom** | **1st symptom** | **2nd symptom** | **3rd symptom** | **4th symptom** | **5th symptom** | **Total** | **%** |
| --- | --- | --- | --- | --- | --- | --- | --- |
| **Confusion** | 39 | 12 | 2 | 0 | 0 | 53 | 22.84% |
| **Drowsiness** | 18 | 14 | 5 | 6 | 1 | 44 | 18.97% |
| **Aphasia** | 10 | 13 | 3 | 1 | 1 | 28 | 12.07% |
| **Dysgraphia** | 4 | 5 | 7 | 3 | 0 | 19 | 8.19% |
| **Disorientation** | 3 | 8 | 4 | 0 | 0 | 15 | 6.47% |
| **Tremor** | 6 | 7 | 0 | 0 | 0 | 13 | 5.60% |
| **Focal deficits** | 4 | 3 | 2 | 0 | 2 | 11 | 4.74% |
| **Agitation** | 1 | 6 | 2 | 1 | 0 | 10 | 4.31% |
| **Headache** | 6 | 1 | 2 | 0 | 0 | 9 | 3.88% |
| **Seizures** | 3 | 3 | 3 | 0 | 0 | 9 | 3.88% |
| **Impaired attention** | 2 | 2 | 1 | 2 | 0 | 7 | 3.02% |
| **Myoclonus** | 2 | 2 | 1 | 0 | 0 | 5 | 2.16% |
| **Cerebellar symptoms** | 2 | 1 | 0 | 1 | 0 | 4 | 1.72% |
| **Coma** | 0 | 1 | 1 | 2 | 0 | 4 | 1.72% |
| **Amnesia** | 0 | 1 | 1 | 0 | 0 | 2 | 0.86% |
| **Total** | **100** | **79** | **34** | **16** | **4** | **233** | **100.00%** |

**Among the 100 patients with ICANS, 233 neurological symptoms were reported. Most frequently reported symptoms are shown in red whereas least significantly reported symptoms appear in green.**

**Supplemental table 3: Comparison of patients with and without severe ICANS**

| **N (%) or Median (IQR)** | **Severe ICANS**  **(Grade 3 or 4)** | **Non-severe ICANS (Grade 1 or 2)** | **P value** |
| --- | --- | --- | --- |
| N | 38 | 62 |  |
| Age (years) | 61 (39 – 68) | 61 (43 – 65) | 0.76 |
| Gender (female) | 17 (44.7%) | 26 (41.9%) | 0.95 |
| SOFA score | 5 (4 – 6) | 4 (3 – 6) | 0.11 |
| ECOG-PS | 1 (1 – 2) | 1 (1 – 2) | 0.54 |
| CFS | 3 (2 – 3) | 2 (2 – 4) | 0.42 |
| CRP (mg/L) | 19 (8 – 54) | 37 (12 – 122) | 0.27 |
| Ferritin (ng/L) | 1808 (890 – 3685) | 1641 (615 – 3530) | 0.27 |
| Platelet count (cells/µL) | 52 000 (29 000 – 79 000) | 63 000 (35 000 – 116 000) | 0.20 |
| Neutrophil count (cells/µL) | 130 (0 – 800) | 630 (35 – 1100) | 0.19 |
| Time from CAR-T cell infusion to ICANS onset (days) | 5 (4 – 8) | 5 (4 – 9) | **0.04** |
| Duration of symptoms (days) | 6 (4 – 11) | 2 (1 – 7) | **0.02** |
| Length of ICU stay (days) | 5 (3 – 8) | 5 (3 – 8) | **0.04** |
| Documented Infection at ICU admission | 3 (8%) | 4 (7%) | 1.00 |
| Cluster 1  Cluster 2  Cluster 3  Cluster 4 | 9 (23.7%)  11 (28.9%)  0 (0)  18 (47.4%) | 21 (33.9%)  8 (12.9%)  15 (24.2%)  18 (29.0%) | **0.002** |

Results are expressed in numbers (%) or median (interquartile range)

CAR-T: chimeric antigen receptor t-cell; CFS: clinical frailty scale; CRP: c-reactive protein; CRS: cytokine release syndrome; ECOG-PS: Eastern Cooperative Oncology Group-Performance Status; ICANS: immune effector cell-associated neurotoxicity syndrome; ICU: intensive care unit; SOFA: sequential organ failure assessment;

**Supplemental table 4: Comparison of patients with isolated ICANS and those with ICANS and CRS**

| **N (%)**  **or Median (IQR)** | Isolated ICANS | ICANS and CRS | *P* |
| --- | --- | --- | --- |
| N | 7 | 93 |  |
| Age (years) | 65 (28, 68) | 60 (45, 67) | 0.650 |
| Gender (female) | 43% | 43% | 0.994 |
| SOFA score | 7 (3-17) | 4 (3-6) | **0.038** |
| ECOG-PS | 2 (1-2) | 1 (0-2) | 0.072 |
| CFS | 3 (2-4) | 2 (2-3) | 0.756 |
| CRP (mg/L) | 3.91 (0.98-21.10) | 44.5 (12.73, 127.75) | **0.025** |
| Ferritin (ng/L) | 3000 (2166-3000) | 1600 (652-3682.5) | 0.304 |
| Platelet count (cells/µL) | 63 000 (37 000-114 000) | 27 000 (14 500-42 500) | **0.016** |
| Time from CAR-T cell infusion to neurological symptoms (days) | 17.42 (6.92-25.81) | 5.25 (3.96-8.16) | **0.002** |
| Duration of symptoms (days) | 16 (11-24) | 4 (1-7) | **0.001** |

Results are expressed in numbers (%) or median (interquartile range)

CAR-T: chimeric antigen receptor t-cell; CFS: clinical frailty scale; CRP: c-reactive protein; CRS: cytokine release syndrome; ECOG-PS: Eastern Cooperative Oncology Group-Performance Status; ICANS: immune effector cell-associated neurotoxicity syndrome; SOFA: sequential organ failure assessment;

**Supplemental Table 5: Imaging exam, electroencephalogram and lumber puncture**

| **Exams** | **N=100 (%), median (IQR)** |
| --- | --- |
| Imaging exam performed at ICANS onset | 90 (90) |
| Normal | 82 (82) |
| Abnormal | 8 (8) |
| Follow-up imaging exam | 52 (52) |
| Normal | 46 (46) |
| Abnormal | 6 (6) |
| Electroencephalogram performed at ICANS presentation | 71 (71) |
| Normal  Nonspecific encephalic dysfunction  Diffuse slowing  Epileptiform discharges | 11 (11)  26 (26)  29 (29)  5 (5) |
| Lumbar puncture performed at ICANS presentation | 48 (48) |
| Normal cerebrospinal fluid | 28 (28) |
| Elevated protein and lymphocytes level | 20 (20) |
| Protein level | 1.4g/L (0.73-2.34) |
| Lymphocyte count | 10 cells/mm^3^ (6-17) |
| Pathogen detected in the CSF (Herpesvirus 6) | 1 (1) |
|  |  |

**Supplemental Table 6: Therapy administered**

| **Therapy administered** | **N=100 (%)** |
| --- | --- |
| Prophylactic anti-seizure therapy | 59 (59) |
| Therapeutic anti-seizure therapy | 22 (22) |
| ICANS treatment plan |  |
| Steroids | 91 (91) |
| Steroids plus anti-IL6 | 66 (66) |
| Steroids alone  Missing data | 25 (25)  9 (9) |
| Type of steroid |  |
| Dexamethasone | 72 (72) |
| Methylprednisolone | 12 (12) |
| Methylprednisolone + dexamethasone | 6 (6) |
| Hydrocortisone | 1 (1) |
| Missing data | 9 (9) |
| Dose of dexamethasone |  |
| 40mg/day | 43 (60) |
| Below 40mg/day | 22 (30) |
| Above 40mg/day | 7 (10) |
|  |  |

**Supplemental Figure 1:**

Heat map showing symptoms’ distribution across clusters. C: Cluster

**C1**

**C3**

**C4**

**C2**
